# Single-cell RNA sequencing shows the immune cell landscape in the kidneys of patients with idiopathic membranous nephropathy

**DOI:** 10.3389/fimmu.2023.1203062

**Published:** 2023-09-04

**Authors:** Manman Shi, Yuxin Wang, Huan Zhang, Zicheng Ling, Xue Chen, Chaojun Wang, Jian Liu, Yuhua Ma

**Affiliations:** ^1^ Department of Nephrology, Traditional Chinese Medicine Hospital of Kunshan, Kunshan, Jiangsu, China; ^2^ Department of Nephrology, Shanghai Changhai Hospital, Navy Medical University, Shanghai, China; ^3^ Department of Internal Medicine, Weiting Community Health Center of Suzhou Industrial Park, Suzhou, Jiangsu, China; ^4^ Department of Nephrology, Ruijin Hospital, Shanghai Jiao Tong University, School of Medicine, Shanghai, China

**Keywords:** single-cell RNA sequencing, idiopathic membranous nephropathy, pro-inflammatory, immune cell, pathogenesis

## Abstract

Idiopathic membranous nephropathy (IMN) is a leading pathological type of the adult primary nephrotic syndrome. Some patients develop end-stage renal disease due to poor response to treatment with steroid and immunosuppressive agents. In order to explore the molecular mechanism of IMN, we collected renal tissue samples from IMN patients and healthy controls and performed analysis by single-cell RNA sequencing (scRNA-seq). A total of 11 kidney cell clusters were identified, including multiple myeloid cell clusters, NK/T cell clusters, and B cell clusters. Most kidney parenchymal and immune cells were enriched in the regulation of immune response, inflammation, fibrosis and endoplasmic reticulum stress. The macrophage population in the IMN group showed a highly activated profile with up-regulated genes related to chemotaxis, inflammation, phagocytosis and fibrosis. CD8+ T cells continued to be cytotoxic in IMN; however, a transition to “inflammageing” GZMK+ CD8+ T cells was observed. The proportion of activated B cells in renal tissues of IMN patients was much higher than that of normal controls, indicating that B cells in IMN might be activated by constant antigenic stimulation. Moreover, the cell-cell interaction analysis revealed the potential communication between renal glomerular cells and immune cells in IMN. Overall, scRNA-seq was applied to IMN to unravel the characteristics of immune cells and elucidate possible underlying mechanisms involved in the pathogenesis of IMN.

## Introduction

Idiopathic membranous nephropathy (IMN) is a renal disease caused by auto-immune disorder and a leading cause of adult primary nephrotic syndrome. In recent years, the incidence of IMN in primary glomerular disease in mainland China has been steadily increasing (1), including younger patients (14 to 44 years old) (2). The response to treatment and disease prognosis of IMN patients are heterogeneous. One-third of untreated IMN patients experience spontaneous remission, another third experience persistent proteinuria, and a final third can develop progressive kidney failure, often in the setting of persistently heavy proteinuria (3).

IMN is an autoimmune glomerular disease. The immune deposit on the glomerular basement membrane is an initiating factor in the pathogenesis of IMN. The identification of target antigens on podocytes, including phospholipase A2 receptor (PLA2R), thrombospondin type 1 domain containing 7A, neutral endopeptidase, Nel-like-1 type molecule and so on, have led to great advancements (4). Due to the lack of immune tolerance, B cells can recognize the above antigens on the podocyte surface and generate specific autoantibodies, forming immune complexes on the glomerular capillary epithelial side (5). After that, the complement system and immune cells in the kidney have a crucial role in IMN pathogenesis, leading to the alteration of podocyte structure, distortion of the slit diaphragm and local immune damage, causing the appearance of substantial proteinuria (6, 7).

Recently, the gradual maturity of single-cell RNA sequencing (scRNA-seq) has made it possible to obtain transcriptomics information in heterogeneous cell populations at the single-cell level (8). scRNA-seq has been successfully applied to a variety of kidney diseases to characterize renal cell populations and the molecular events involved in the disease (9). Studies of multiple kidney diseases using scRNA-seq technologies transformed our understanding of disease pathogenesis, including IMN. Currently, two researches about scRNA-seq of IMN found that differentially expressed genes (DEGs) in renal parenchymal cells of IMN patients were genes mainly concentrated on regulation of inflammation and immunity, including chemokines, HLA-related genes, IL-17 signaling, TNF signaling, NOD-like receptor (NLR) signaling and MAPK signaling (10, 11). However, studies on the detailed analysis of immune cell subsets in IMN renal tissue are still lacking.

Herein, we applied scRNA-seq technology to explore transcriptome data, intercellular communication and molecular events of kidney parenchymal and immune cells from patients with IMN and normal controls in order to find the potential pathogenesis contributing to the disease development and regulatory mechanisms of IMN.

## Results

### Study design and single-cell transcriptomic data

**Figure 1:**
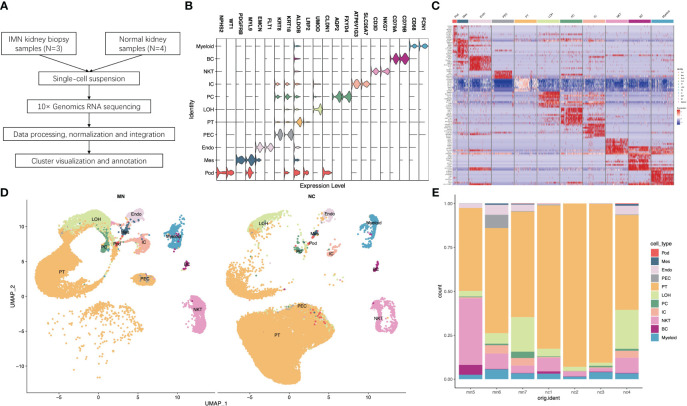
Single-cell RNA sequencing reveals the kidney cell populations in IMN and control subjects. **(A)** Overview of single-cell RNA sequencing and data processing. **(B)** Violin plot of marker genes that identified the eleven distinct cell clusters. **(C)** Heat map showing marker genes of each cell cluster. **(D)** Eleven distinct cell clusters identified by UMAP plotting from three IMN patients and four normal controls. **(E)** Bar plots represent the proportion of cell clusters in each subject. IMN, idiopathic membranous nephropathy; Pod, podocytes; Mes, mesangial cells; Endo, endothelial cells; PEC, parietal epithelial cells; PT, proximal tubule cells; LOH, loop of Henle cells; PC, principal cells; IC, intercalated cells; NKT, natural killer cells and T cells; BC, B cells; Myeloid, myeloid cells.

In order to generate a detailed atlas of the kidney cells in patients with IMN, we comprehensively analyzed the transcriptional data from three IMN patients and four healthy controls (data for one case were our data, and the other three cases were obtained from the Gene Expression Omnibus (GEO) database (GSE131685) (12)) (**Figure 1A** and **Table S1**). All three IMN patients presented with nephrotic syndrome, with proteinuria ranging from 4.35 to 15.25 g per 24 h and serum albumin ranging from 14.5 to 28.8g/L. The estimated glomerular filtration rate calculated by Chronic Kidney Disease Epidemiology Collaboration equation of IMN patients were 100.75 to 124.18 ml/min/1.73m^2^. None of IMN patients had coexisting diseases or secondary kidney diseases. The healthy control from our own database was a 59-year-old male without proteinuria or hematuria, and didn’t have any chronic diseases like hypertension and diabetes.

For each sample, scRNA profiling was performed using 10× Genomics sequencing. After data processing and quality control, 48573 cells were kept for downstream normalization, principal component analyses (PCA), and harmony integration. In order to find the gene expression features of each cluster, we performed differential expression analysis and clarified 11 cell types based on marker genes (**Figure 1B**). Mesangial cells were labeled by PDGFRB and MYL9; podocytes were labeled by NPHS2 and WT1, while endothelial cells were labeled by EMCN and FLT1. ALDOB and LRP2 were defined as specific markers for proximal tubule cells, whereas CLDN1 and UMOD were specially expressed in the loop of Henle. The traditional marker genes AQP2 and FXYD4 defined principal cells, ATP6V1G3 and SLC26A7 defined intercalated cells, and KRT18 and KRT8 defined parietal epithelial cells (PEC). Besides, expression of CD68 and FCN1 were considered as myeloid cells, and high expression of NKG7 and CD3D described natural killer and T (NK/T) cells. For B cells, we used CD79A and CD79B as marker genes. The detailed cell-specific marker genes of kidney parenchymal cells and immune cells are shown in **Table 1**. The top 10 genes with the highest expression in each cell population are depicted in **Figure 1C**. As seen in **Figure 1D**, the visualization of cell cluster was implemented through uniform manifold approximation and projection (UMAP). The proportion of cell populations varied among different kidney samples (**Figure 1E**).

**Table 1:** Cell-type-specific gene markers of different cell types.

Cell Type	Abbreviation	Gene Markers
Podocytes	Pod	NPHS2, WT1
Endothelial Cells	Endo	EMCN, FLT1
Mesangial Cells	Mes	PDGFRB, MYL9
Parietal Epithelial Cells	PEC	KRT18, KRT8
Proximal Tubule Cells	PT	ALDOB, LRP2
Loop of Henle	LOH	CLDN1, UMOD
Principal Cells	PC	AQP2, FXYD4
Intercalated Cells	IC	ATP6V1G3, SLC26A7
Myeloid Cells	Myeloid	CD68, FCN1
NK/T cells	NK/T	CD3D, NKG7
B cells	BC	CD79A, CD79B

### Identification of DEGs and enrichment analysis in different kidney parenchymal cells in patients with IMN

To investigate the gene expression changes in glomerular cells and renal tubular cells of IMN patients, we performed DEG analysis and Gene Ontology (GO) enrichment analysis on single-cell transcriptome data of IMN patients and healthy controls (**Tables S2**, **S3**). The results showed that VCAM1 was one of the most up-regulated genes in podocyte of IMN patients (**Table S2** and **Figure S1A**), which plays a central role in leukocyte recruitment during inflammation and is essential for leukocyte adhesion (13). Besides, GO enrichment analysis showed that DEGs in podocytes were enriched in the WNT signaling pathway (**Table S3** and **Figure S2**), which is involved in fibrosis, podocyte dedifferentiation, and mesenchymal transition (14). The DEGs of mesangial cells were enriched in biological processes such as extracellular matrix organization, cell-matrix adhesion and cell chemotaxis (**Table S3** and **Figure S2**). Glomerular endothelial cells of IMN patients highly expressed RPS4Y1 (**Table S2** and **Figure S1C**), a member of the S4E family of ribosomal proteins, and previous studies have found that RPS4Y1 can promote inflammation and apoptosis of endothelial cells by activating the phosphorylation of p38, leading to endothelial cell dysfunction (15). MHC-II genes such as HLA-DRB5 and HLA-DRB1 were also highly expressed in IMN endothelial cells (**Table S2** and **Figure S1C**). Correspondingly, GO enrichment analysis results confirmed that endothelial DEGs were enriched in the synthesis and binding of MHC-II molecules (**Table S3** and **Figure S2**). Comparison of DEGs in PEC revealed enrichment of genes involved in ribonucleoprotein complex biogenesis, RNA splicing, ribosome biogenesis, and cytoplasmic translation (**Table S3** and **Figure S2**). Compared with the control group, the DEGs of PT cells in IMN group were enriched in generation of precursor metabolites and energy, small molecule catabolic process and cellular amino acid metabolic process (**Table S3** and **Figure S2**). Besides, PT of IMN patients highly expressed DEFB1 (**Table S2** and **Figure S1E**), suggesting that PT may be involved in inflammatory response and chemotaxis to immune cells. LOH cells highly expressed CANX, HSP90B1, TMBIM6 and MAN1A1, which helps to participate in response to endoplasmic reticulum stress (**Table S3** and **Figure S2**). DEGs of PC were enriched in GABARAP, CTNNB1 and TBL1XR1, which involved in the proteasome-mediated ubiquitin-dependent protein catabolic process, and enrichment of ATP1B1, STC1, HSD11B2 and EGR1 were involved in the process of response to hypoxia (**Table S3** and **Figure S2**). The DEGs of IC between IMN patients and healthy controls were enriched in response to endoplasmic reticulum stress, endoplasmic reticulum-associated degradation (ERAD) pathway and response to topologically incorrect proteins, of which TMBIM6, AMFR, CLU, TMED2, HSP90B1, CALR and CANX were involved (**Table S3** and **Figure S2**). In addition, DEGs for antigen processing and presentation were enriched in ICs of IMN patients, including HLA-A, HLA-B, HLA-C, and HLA-E for MHC-class I molecules and HLA-DRB1 for MHC-class II molecules (**Tables S2**, **S3** and **Figures S1H**, **S2**).

### The immune microenvironment of IMN exhibits pro-inflammatory traits

Next, we focused on cells of the kidney immune microenvironment. GO analysis showed that myeloid cells, NK/T cells and B cells of IMN patients all presented with immune activation. Myeloid cells of IMN highly expressed chemokines encoding genes like CCL4, CCL4L2, CCL3, CCL3L1 and CXCL2, MHC-class II genes like HLA-DRB5, HLA-DRB1 and HLA-DQA1 and genes of inflammatory cytokines like IL1B and TNF (**Table S2** and **Figure S1I**), which were associated with leukocyte cell-cell adhesion, myeloid cell differentiation, regulation of T cell activation and MHC protein complex assembly (**Table S3** and **Figure S2**). Besides, GO analysis showed that DEGs of NK/T cells of IMN were enriched in lymphocyte differentiation and T cell receptor signaling pathway, while B cells of IMN were enriched in B cell activation, lymphocyte differentiation and immune response-regulating signaling pathway (**Table S3** and **Figure S2**).

Then, we identified myeloid cells, B lymphocytes, and T lymphocytes to further subset and analyze different cell clusters of steady and activated macrophages, plasmacytoid dendritic cells (pDCs), classical dendritic cells (cDCs), CD4+ T cells, CD8+ T cells, double-negative T (DNT) cells, natural killer T (NKT) cells, natural killer (NK) cells, naïve B cells, and memory B cells based on typical marker genes and gene signatures. Based on this, we noted quantitative shifts of pro-inflammatory traits in the cellular composition of the IMN kidney immune microenvironment compared to normal kidney tissue.

### Classification and annotation of five myeloid cell clusters

**Figure 2:**
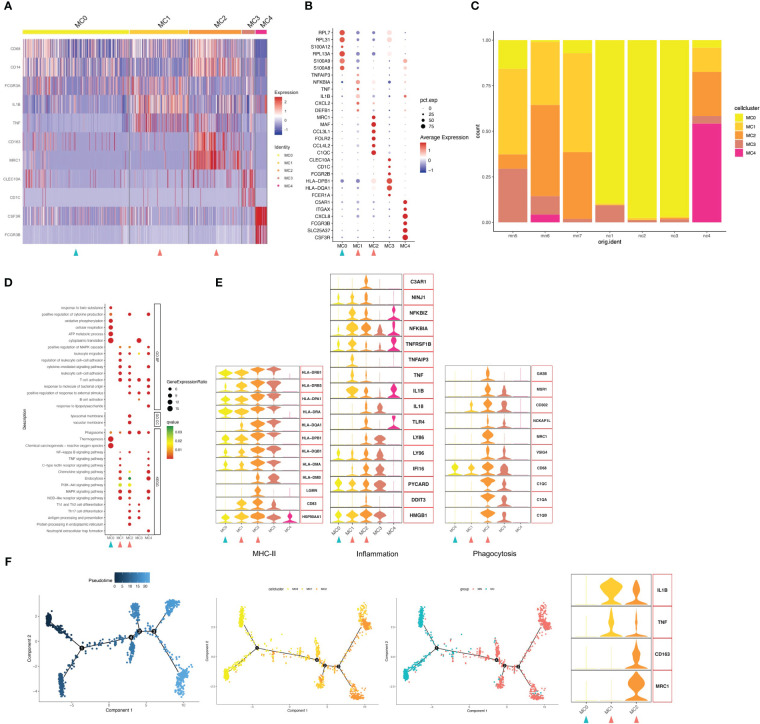
Myeloid cells have five subgroups. **(A)** Heatmap of feature genes that clustered each subgroup. **(B)** Dotplot demonstrates the relative gene expression and percentage of highly expressed genes in each cell group. The color of the dots represents the relative expression levels of genes, while the size of the dots represents the proportion of cells expressing the gene. **(C)** Barplot denoting the percentages of five myeloid cell subgroups in each sample. **(D)** Dotplot showing the GO and KEGG pathway enrichment analysis of five myeloid cell subgroups. **(E)** Violin plots of gene signatures related to MHC-II, inflammation and phagocytosis in different subgroups. **(F)** Pseudotime cell trajectory analysis on monocytes/macrophages. We correlated the cell trajectory analysis with the three clusters of steady-state kidney monocytes (MC0), inflammatory macrophages (MC1) and alternatively activated (M2-like) macrophages (MC2). Green and red arrowheads indicate clusters mainly composed of controls and IMN patients, respectively.

Focused analysis of the 1671 cells in the myeloid cluster revealed 5 finer clusters (clusters MC0–MC4, **Figure 2A** and **Figure S3**). Cluster MC3 was closest to CD1C+ DCs, which highly expressed CD1C as markers of the cDC cell population, and MHC class II genes such as HLA-DPB1 and HLA-DQA1 were also highly expressed (**Figures 2A, B**). We used GO and Kyoto Encyclopedia of Genes and Genomes (KEGG) annotation to compare the GO terms of macrophages in different macrophage clusters. GO and KEGG pathway analysis indicated that highly expressed genes (HEGs) of MC3 were involved in B cell activation, T cell activation and subclass differentiation, and antigen presentation. Cluster MC4 cells were most similar to neutrophils, with characteristically expressed genes of CSF3R, FCGR3B, and CXCL8, and involvement of neutrophil extracellular trap formation and LPS response based on GO and KEGG pathway enrichment analysis. Cluster MC1 cells were most similar to CD16+ monocyte with high expression of CD16 (FGCR3A), while MC2 were similar to CD14+ monocyte. Interestingly, we found that MC1 and MC2 clusters mostly presented in IMN kidneys, while the proportion was very low in the control kidney. We therefore speculated that MC1 and MC2 may represent infiltrating monocyte/macrophage subsets (**Figure 2C**). Finally, MC0 had low expression of CD14 and CD16, while up-regulated expression of ribosome-related genes RPL7, RPL31, and RPL13A, and classic monocyte markers S100A8 and S100A9 was observed. Besides, GO analysis showed that MC0 HEGs were significantly involved in cytoplasmic translation, ATP metabolic process, cellular respiration, and oxidative phosphorylation. Since MC0 constituted a majority of myeloid cells in normal kidneys, it likely represented steady-state monocytes.

Next, we analyzed the gene expression pattern to obtain functional features of each cell population (**Figures 2D, E**). MC2 expressed up-regulated levels of genes encoding phagocytic receptors such as CD163, CD68, FOLR2 and MRC1, which suggested representing alternately activated (M2-like) macrophages. Besides, MC2 showed up-regulated expression of C1Q, such as C1QA, C1QB and C1QC, which plays a role of opsonization and enhances removal of apoptotic cell debris by upregulating the expression of GAS6, VSIG4, MSR1, MRC1 and HLA. C1QA/C1QB/C1QC are specific marker genes for kidney-resident macrophages (KRMs) (16); however, the role of C1Q+ KRMs in disease status was not fully understood. Our findings suggested that KRMs in IMN patients were highly activated and secreted pro-inflammatory factors to participate in local immune damage. GO and KEGG analysis showed that terms in MC2 were mainly enriched in lymphocyte adhesion, cytokine production, Th1/2 and Th17 cell differentiation, antigen presentation, and inflammatory processes including NF-κB, MAPK, and NLR signal pathways.

Cluster MC1 was found to express the highest level of inflammation-related genes, such as IL1B, NFKBIA, TNF and TNFAIP3. GO and KEGG pathway analysis also suggest that MC1 is involved in processes related to inflammatory response, such as NLR signaling pathway, MAPK signaling pathway, chemokine signaling pathway, C-type lectin receptor signaling pathway and TNF signaling pathway. Besides, the present study found that the WNT signaling pathway was up-regulated in the MC1 population, and its overactivity could promote renal fibrosis.

Trajectory analysis suggested possible transitions among the three monocytes-macrophages cell populations, with MC1 linking MC0 and MC2 (**Figure 2F**). Besides, as MC0 were the most similar to peripheral blood CD16+ monocytes, it was assumed that they progressed from monocytes (MC0) to inflammatory macrophages (MC1) and then an M2-like phenotype (MC2). Actually, we discovered that MC1 expressed up-regulated inflammation-related genes, while MC2 highly expressed both phagocytosis and inflammatory-related genes. However, unlike the other two types, MC0 expressed neither inflammation nor phagocytosis-related genes. Furthermore, we found a gradually increased expression of MHC-II genes through the transition from MC0 to MC2.

Among myeloid cell types in the IMN kidneys, activated macrophages, including inflammatory and phagocytic macrophages (MC1 and MC2), were increased, while steady-state kidney monocytes (MC0) were decreased. The monocyte-macrophages in the NC group were mainly composed of the MC0 population representing steady-state monocytes, while those in the MN group were mainly composed of MC1, mediating inflammation and fibrosis, and MC2, mediating inflammation and phagocytosis. These findings suggested that macrophages in MN patients are activated, and inflammatory CD16+ macrophages differentiate into a state of M2-like cells in the tissue.

### NK/T cells contain ten clusters of NK, NKT, CD8+ T, CD4+ T, and DNT cells

**Figure 3:**
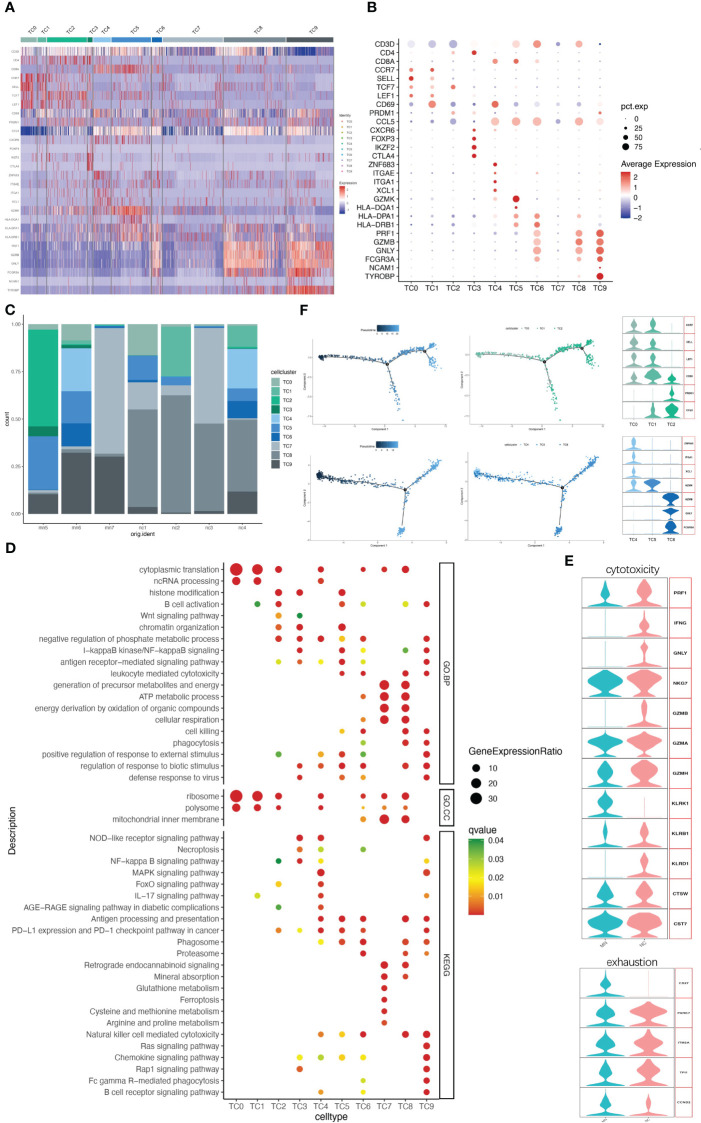
NK/T cells have ten subgroups. **(A)** Heatmap of feature genes that clustered each subgroup. **(B)** Dotplot demonstrates the relative gene expression and percentage of highly expressed genes in each cell group. The color of the dots represents the relative expression levels of genes, while the size of the dots represents the proportion of cells expressing the gene. **(C)** Barplot denoting the percentages of ten NK/T cell subgroups in each sample. **(D)** Dotplot showing the GO and KEGG pathway enrichment analysis of ten NK/T cell subgroups. **(E)** Violin plots of gene signatures related to cytotoxicity and exhaustion in different subgroups. **(F)** Pseudotime cell trajectory analysis on T cells. We correlated the cell trajectory analysis with the three CD4+ clusters of naive CD4+ T cells (TC0), central memory CD4+ T cells (TC1), and effector memory CD4+ T cells (TC2) (upper), and three CD8+ clusters of resident memory CD8+ T cells (TC4), GZMK+ CD8+ T cells (TC5), and cytotoxic T lymphocyte (TC6) (lower).

IMN kidneys comprised 3062 T cells and NK cells, which were separated into ten cell clusters of CD4+ T cells, CD8+ T cells, NK cells, NKT cells and DNT cells (**Figures 3A–C** and **Figure S4**). Cluster TC9 contains CD16+ NK cells, which was characterized by high expression of marker genes CD16 (FGCR3A), CD56 (NCAM1) and DAP12(TYROBP), and high level of cytotoxic genes such as PRF1, GZMB, and GNLY, while lacking CD3D, the marker gene of T cells. The TC6 cell cluster expressed a similar level of cytotoxic genes, along with high expression of CD3D and CD8A, indicating a cytotoxic T lymphocyte (CTL) identity, and the NKT cell cluster TC8 was, characterized by co-expression of the T cell marker CD3D and the NK cell marker FCGR3A and GNLY, with no expression of CD8A (**Figures 3A, B**). Correspondingly, CD16+NK cells (TC9), CTL (TC6) and NKT (TC8) were primarily involved in phagocytosis, proteasome, cell killing, natural killer cell-mediated cytotoxicity and antigen processing and presentation (**Figure 3D**).

A second population of CD8+ T cells, other than CTL, was TC5. This cell population characteristically expressed high level of GZMK but lacking GZMB, GNLY and PRF1, while presenting high level of HLA-DR/DP/DQ (**Figures 3A, B**), consistent with earlier reports (17, 18). A third CD8+ T cell population (cluster TC4) was characterized by ZNF683 (HOBIT), ITGAE, ITGA1, XCL1, and CD69, indicating resident memory T cells. CD8+ T cells might present with exhaustion markers in some patients with autoimmune-related kidney diseases, like SLE and IgAN (19, 20). However, no such changes occurred in CD8+ T cells in our data. Three CD8+ T cells clusters highly expressed cytotoxic marker genes and rarely expressed typical exhaustion genes like PD-1 (PDCD1), CTLA4 and BTLA in both IMN patients and normal controls (**Figure 3E**).

TC7 is another cell cluster expressing CD3D but without CD4, CD8A, or NK cell-associated maker genes expression, which was recognized as a DNT cell. DNT cells are an unconventional kind of T cells with neither CD4 nor CD8 expression, comprising 1-3% of circulating T cells of healthy persons (21). They are also found in greater quantities in normal kidney tissue (22). DNT cells have been considered as a pathogenic factor in various autoimmune diseases (23). Here we compared DNT cells with other NK/T cell populations, including CD4+ T, CD8+ T, NKT, and NK cells, to analyze the expression profile of DNT cells. A total of 80 genes expressed by DNT cells were significantly higher than other T cells, among which the top 5 genes were MT1G, MIOX, MT1H, ATP5F1E, and FXYD2, representing genes related to mitochondrial and energy metabolism (**Table S4**). GO and KEGG pathway enrichment analysis revealed that HEGs in DNT cells were enriched in the generation of precursor metabolites and energy, ATP metabolic process and respiration cellular respiration while lacking immune processes (**Figure 3D**).

Clusters TC0, TC1, TC2, and TC3 contained CD4+ T cells (**Figures 3A, B**). Cluster TC3 highly expressed FOXP3 and IKZF2 (HELIOS), suggesting the representation of T regulatory (Treg) cells. Cluster TC2 mainly expressed PRDM1 and CCL5, representing primarily effector memory CD4+ T cells (CD4+Tem). Both clusters TC0 and TC1 consisted of cells that mostly expressed CCR7, SELL and TCF7, but CD69 expression differed between the two cell populations. TC1 highly expressed CD69, which was classified as central memory CD4+ T cells (CD4+Tcm), while TC0 with low CD69 expression tended to be naive CD4+ T cells (CD4+Tn). It has been reported that helper T cell (Th) populations also plays an important role in the immune disorder of IMN (24). However, our data did not identify those Th-subset cells including Th2, Th17 and follicular helper T (Tfh) cells.

Clusters TC2 and TC3 of the CD4+ T cell population were mainly distributed in IMN patients, while TC0 and TC1 were mainly distributed in the normal control group (**Figure 3C**). GO and KEGG pathway enrichment analysis indicated that the HEGs of clusters TC0 and TC1 are mainly involved in cytoplasmic translation, ncRNA processing and ribosome, while TC2 and TC3 are mainly involved in immune and inflammation processes like B cell activation, antigen-receptor mediated signal pathway, NLR, WNT, NF-κB signal pathway, and PD-1 checkpoint pathway (**Figure 3D**).

### Analysis of B-cell clusters

**Figure 4:**
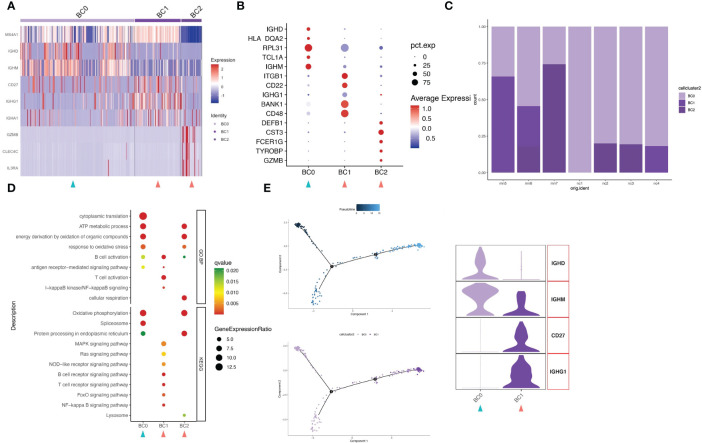
B cells have three subgroups. **(A)** Heatmap of feature genes that clustered each subgroup. **(B)** Dotplot demonstrates the relative gene expression and percentage of highly expressed genes in each cell group. The color of the dots represents the relative expression levels of genes, while the size of the dots represents the proportion of cells expressing the gene. **(C)** Barplot denoting the percentages of three B cell subgroups in each sample. **(D)** Dotplot showing the GO and KEGG pathway enrichment analysis of three B cell subgroups. **(E)** Pseudotime cell trajectory analysis on B cells. We correlated the cell trajectory analysis with the two clusters of naive (BC0) and memory B cells (BC1). Green and red arrowheads indicate clusters mainly composed of controls and IMN patients, respectively.

Upon further analysis, 310 B cells were divided into 3 different B-cell subtypes (clusters BC0-BC2). Highly expressed activation markers like CD27, CD48, BANK1 and ITGB1 provided an activated identity of cluster BC1. In addition to that, high level expression of immunoglobulin genes IGHG1 and IGHA1 yet lack of IGHD and IGHM help to define BC1 as memory B cells (**Figures 4A, B**; **Figure S5**). Cluster BC0 were the major subset (63.2%, 196 cells) of B cell populations (**Figure 4C**). The HEGs of BC0 included IGHD, IGHM, TCL1A and B cell markers CD20 (MS4A1), demonstrating up-regulation of genes typical of naive B cells. GO and KEGG pathway analysis revealed that HEGs of BC0 cells are involved in ATP metabolic process, oxidative phosphorylation, spliceosome, and protein processing (**Figure 4D**). Our results further suggest BC0 as naive B cells, which maintain normal cellular biological processes such as energy, metabolic process and protein synthesis, but not in the state of immune activation. Cluster BC2 expressed marker genes of pDCs, including GZMB, CLEC4C and CD123 (IL3RA). Within the IMN B lymphoid cell compartment, memory B cells (BC1) were increased, while naïve B cells (BC0) were decreased compared to normal tissue controls.

### Trajectory analysis reveals enhanced immune activation in IMN

In order to understand the underlying evolvement of cellular status, we applied the pseudo-time cell trajectory to analyze various lymphocytes in kidney, including CD8+ T cells, CD4+ T cells and B cells. Interestingly, we discovered the transition of CD8+T cells from memory type (TC4) towards the subpopulations of GZMK positive (TC5), with the intermediate state of CTL (TC6) (**Figure 3F**). GZMK+CD8+ T cells can contribute to an ‘inflammageing’ phenotype (25), and this cluster is more frequently seen in IMN patients (16.2% in IMN vs. 8.1% in the normal control group). Such inflammageing state was supported by the downregulation of PRF1, GZMB, GNLY, and FCGR3A in cell cluster TC5. CD4+T cells also showed the transition from a naïve state (CD4+Tn and CD4+Tcm) toward a more immunologically mature state (CD4+Tem) (**Figure 3F**). Regarding the B cells, they were mainly naïve B cells (BC0) and memory B cells (BC1), as indicated by HEGs of IGHM (BC0) and IGHG1 (BC1), respectively (**Figure 4E**). In sum, we characterized the transition of immune cells to a more immunoreactive status in renal tissues of IMN patients with unique gene expression markers. These results suggested the possibility of enhanced immune activation and inflammatory landscape in IMN.

### Cell-to-cell ligand-receptor interactions

**Figure 5:**
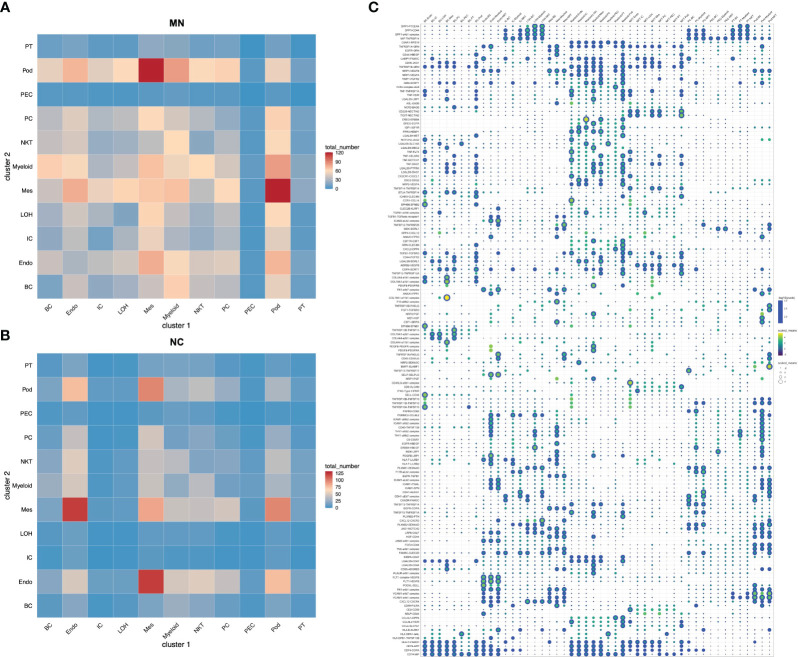
Cell-cell interactions of ligands and receptors between different kidney cell types. Heatmap showing ligand-receptor interactions between kidney cell clusters in **(A)** IMN patients and **(B)** control subjects. **(C)** Dot plot showing interactions between kidney parenchymal cell clusters and immune cell clusters. BC, B cells; Endo, endothelial cells; IC, intercalated cells; LOH, loop of Henle; Mes, mesangial cells; Myeloid, myeloid cells; NKT, natural killer cells and T cells; PC, principal cells; PEC: parietal epithelial cells; Pod, podocytes; PT, proximal tubule cells.

To identify the potential interaction between renal parenchymal and immune cells, we performed ligand-receptor analysis using cellphoneDB. Compared with normal controls, the cell-cell interactions in IMN patients were more complex (**Figures 5A, B**). The potential interactions between immune cells and renal parenchymal cells mostly involved chemokines, adhesion molecules and TNF superfamily, which played a role in chemotaxis, adhesion, immune cell regulation and inflammatory response. The most prominent interactions occurred in podocytes, mesangial cells, endothelial cells versus myeloid cells, B cells and NK/T cells. CXCL12, MIF, ICAM1, VCAM1 expressed in podocytes, mesangial cells and endothelial cells promoted myeloid cells, B cells and NK/T cells chemotaxis through the receptors CXCR4, TNFRSF14 and ITGAL, and express TNFRSF1A, TNFRSF12A and TNFRSF14 as well to interact with TNF, TNFSF13 and TNFSF14 in immune cells, which may participate in inflammation response. The detailed information on cell-cell interactions was all shown in **Figure 5C**.

## Discussion

The present study provided important insights into the single-cell transcriptional signatures of IMN and controls. Here we characterized the renal parenchymal cell and immune cell profiles of IMN renal tissues and discovered the distinct expression signatures of myeloid cell, T cell and B cell subsets. First, we found that DEGs in kidney parenchymal and immune cells were enriched in the regulation of immune response, inflammation, fibrosis and endoplasmic reticulum stress. Second, evidences were discovered for an increased proportion of inflammatory and M2-like macrophages and cell state transition of inflammatory macrophages towards M2-like cells. Besides, inflammatory macrophages highly expressed the genes of the WNT signaling pathway, suggesting a possibility of pro-fibrotic effects. Third, CD8+ T cells were lacking in exhaustion markers, indicating that they still have cytotoxic effects in IMN. Then, we identified the presence of GZMK+CD8+ T cells and the transition of CTL to GZMK+CD8+ T cells. The proportion of GZMK+CD8+T cells in IMN was higher, suggesting greater levels of “inflammageing” in these patients. Besides, a transition of state was presented from naïve B to activated B cells, and compared to normal controls, activated B cells accounted for a higher proportion in IMN patients, indicating B cells in IMN might be activated by constant antigen stimulation. Finally, the cell-cell interaction analysis revealed the potential communication between renal glomerular cells and immune cells in IMN, which infers the glomerular parenchymal and immune cells play important roles in IMN.

Podocytes are the major targets of IMN, as the binding of antibodies to them form immune deposits and lead to subsequent inflammation and immune response (26). Here we analyzed and compared the molecular differences of podocyte between IMN and normal control, and found that VCAM1 to be one of the most up-regulated genes. VCAM-1 is important for leukocyte chemotaxis and adhesion (13). Under normal circumstances, there is a small amount of VCAM-1 expression in podocytes; however, the expression of VCAM-1 in podocytes is significantly up-regulated by the stimulation of TNF-α (27). Our study revealed that podocytes in IMN may promote the infiltration of inflammatory cells and local immune activation by up-regulating the expression of VCAM1. Endothelial cell and tubulointerstitial injury can also be seen in some IMN patients (26, 28). In our study, we discovered that DEGs of endothelial cells and IC are related to MHC molecules, which suggests them participating in the antigen presenting progress. These are consistent with a previous single-cell sequencing study of IMN, which showed enrichment of inflammation and immune regulatory genes in kidney parenchymal cells (11).

IMN is a kidney-specific autoimmune disease. Various immune cells such as B cells, T cells and macrophages infiltrated renal tissue (29–31). A single-cell sequencing study of IMN kidney tissue identified six leukocyte clusters consisting of dendritic cells, macrophages, monocytes, mast cells, plasma cells, and T cells, among which macrophages were reported to highly express genes responsible for regulating leukocyte activation, inflammatory responses and are involved in antigen processing and presentation (11). However, other immune cells were not analyzed for meaningful differences due to insufficient numbers or other reasons (11). In a different study, which included one MN and one healthy donor, single-cell transcriptome analysis of kidney tissue identified immune cells including macrophages, NK cells, NKT cells, and neutrophils (10). Similarly, this study found that macrophages in the MN group were activated, with high expression of CXCL2, CXCL3, CXCL8, FPR3, CCL2 and NR4A2 (10). Besides, glomerular M2-like macrophage infiltration was reported to be associated with IgG and complement deposition in renal tissue of IMN patients (29).

In order to further analyze the changes of immune cells in IMN patients and their role in disease progression, this single-cell sequencing study we conducted focusing on immune cells in IMN kidney tissue. Our analysis identified and provided a detailed description of kidney immune cells such as myeloid cells, NK/T cells and B cells. We first compared the DEGs of B cells, NK/T cells and myeloid cells between NC and IMN, and found that myeloid cells in IMN patients highly expressed chemokines such as CCL4, CCL4L2, CCL3, CCL3L1, CXCL2 and CXCL3, as well as inflammatory factors such as IL1B and TNF. Then we divided myeloid cells include monocytes, macrophages and DCs. Two types of macrophages are present in normal renal tissue: tissue-resident macrophages and macrophages differentiated from circulating monocytes. Macrophages and their products can make a contribution to kidney injury and repairment, including the progression from kidney inflammation to renal fibrosis. In the acute inflammatory phase of the disease, infiltration of macrophages are mainly M1-like, while macrophages transit to an M2-like phenotype during the chronic phase of the disease (32). As we did not identify the M1-like macrophage, macrophages of three subclasses in our study were found to transit from steady-state monocytes towards an inflammatory type and finally to M2-like macrophages.

Macrophages have a key role in maintaining renal homeostasis as macrophages induced by dysregulated signals can drive disease processes such as inflammation and fibrosis (33). Macrophage infiltration is a common feature of active fibrosis in chronic diseases (34). Additionally, macrophages can promote extracellular matrix synthesis and deposition as key inflammatory cells, and release inflammatory cytokines, transforming growth factor β (TGF-β) and Wnt as well to cause renal fibrosis (35, 36). Wnt/β-catenin signaling plays a key role in renal inflammation and fibrosis, as activation of Wnt/β-catenin signaling pathway can induce macrophage chemotaxis, pro-inflammatory cytokines secretion and extracellular matrix expression (14, 37). Overactivity of Wnt/β-catenin in macrophages can induce proliferation and chemotaxis of macrophages and transform into M2-like macrophages, eventually causing kidney fibrosis (35). However, the current study discovered that the WNT signaling pathway was up-regulated in the MC1 population representing inflammatory macrophages but not the MC2 population representing M2-like macrophages. In contrast, M2-like macrophages mainly expressed genes related to inflammation and phagocytosis.

T cell populations found in kidney tissue comprised a variety of subclasses. For CD4+ T cells, we identified CD4+Tn, CD4+Tcm, CD4+Tem and Treg; no helper T cells such as Th1, Th2, Th17 and Tfh were detected, indicating that the infiltration of T cells in IMN kidney may not be dominated by T helper cells. The discovery of three CD8+ T-cell subtypes, which underwent a CTL-to-GZMK+CD8+ T cell transition, and the abundance of GZMK+CD8+ T cells in IMN kidney tissue, suggested that CD8+T cells in IMN were more likely to be “inflammageing”. We also classified another T cell, i.e., DNT cell, which was identified as a distinct subtype of T cells without expression of CD4, CD8 or NK cell markers, accounting for 22.3% and 15.7% of total T cells in IMN and controls, respectively. DNT cells have been reported as an important participant in the pathogenesis of multiple autoimmune diseases and the maintenance of immune homeostasis (23, 38). The function of DNT cells is quite complex, which means DNT cells may play a pro-inflammatory or anti-inflammatory role under different physiological and pathological conditions (39, 40). However, in the present study, we did not find such functions mentioned above in DNT cells, which suggested that DNT cells, similar to CD4+T cells, were not the main cells that have a key role in the pathogenesis and progression of IMN.

Moreover, we found that B cells present with two states, naive and activated cells. Noteworthy, although there was little difference in the number of B cells in the IMN and control samples, naïve B cells were mainly distributed in the control group, while memory B cells were almost exclusive in the IMN group, which was consistent with the pathogenesis of antigen-stimulated activation of B cells in patients with IMN.

We determined cell-cell interactions among kidney parenchymal and immune cell clusters with ligand-receptor crosstalk analysis. Results showed that ligand-receptor pairs about chemotaxis, inflammation and immune regulation were observed in IMN. We found podocytes, mesangial cells and endothelial cells expressed chemokines like CXCL12 and adhesion molecules like ICAM1 and VCAM1, which interacted with their receptors CXCR4 and ITGAL expressed in immune cells, implying renal glomerular parenchymal cells may serve as a modulator for immune cells recruitment and infiltration. Besides, podocytes, mesangial cells and endothelial cells also expressed TNF superfamily receptors and interacted with TNF, TNFSF13 and TNFSF14 in immune cells, suggesting the inflammatory response caused by crosstalk from immune cells to parenchymal cells. Abundant intercellular communication in kidney cells of IMN serves as a potential therapeutic target and deserves further investigation in the future.

There were several limitations in the present study. First, the sample size included in this study is small. Second, few important glomerular cells such as podocytes and mesangial cells are identified for technical reasons. At last, results in this study are at the transcriptome level. Therefore, the reported findings need to be validated by populations with larger sample size, and further verified by histological and functional experiments using cell lines and animal models.

Overall, the present study showed cell-specific transcriptional profiles and signaling pathways involved in the kidney in IMN patients. In addition, scRNA-seq of kidney immune cells revealed new insights into the immune disorder and pathogenesis of IMN, which could provide opportunities for developing new therapies.

## Methods

### Study design and renal specimens preparation

Renal specimens of three IMN patients with tissue-positive PLA2R were obtained from the nephrology department in the Traditional Chinese Medicine Hospital of Kunshan by renal biopsy. The control renal specimen was collected by excision of paracancerous tissue at least 2 cm away from tumor tissue in a renal cell carcinoma patient, who underwent radical nephrectomy from the urology department in the Traditional Chinese Medicine Hospital of Kunshan. Fresh renal tissues were washed with sterile saline solution after collection, and kept in MACS Tissue Storage Solution at 4°C.

### Single-cell suspension and preprocessing of scRNA-Seq data

For single cell isolation, renal samples were cut into 0.5mm^2^ pieces and then transferred to a 1.5mL-tube containing 1ml collagenase II and collagenase IV mixtures for digestion, subsequently filtered to remove cell debris and impurities. Next, 1 ml Red Blood Cell Lysis was added and incubated on ice for 2-10 min to lyse remaining red blood cells. After incubation, the cell suspension was centrifuged at 300g for 5 min at room temperature and then resuspended in 100 μl Dead Cell Removal MicroBeads. Subsequently, we removed dead cells using Dead Cell Removal Kit and resuspended cell samples in 1× PBS (0.04% BSA). Finally, cell suspension was centrifuged at 300 g for 3 min at 4°C and resuspended in 50 μl of 1× PBS (0.04% BSA). The overall cell viability was confirmed by trypan blue staining. The subsequent processing was performed if the cell viability was > 80%, with background debris <50% and clumping rate < 10%. The qualified cells were washed and resuspended for a suitable cell concentration of 700~1200 cells/ul.

The single-cell transcriptomic amplification and library preparation after single-cell suspension loaded onto the microfluidic chip were performed according to the manufacturer’s instructions for 10×Genomics single-cell 3’ kit (V3). The following mRNA trapping, reverse transcription, cDNA purification and amplification were prepared according to the manufacturer’s protocol. The constructed library was sequenced using a Novaseq 6000 system.

### Statistical analysis of scRNA-Seq data

Raw data from three IMN patients and one normal control from the sequencer was first processed by CellRanger (version 3.0) to get the FASTQ files, which were then aligned to the human genome reference sequence GRCh38. Finally, a collection of files, including barcodes, gene features and gene expression matrix, was obtained through CellRanger. The scRNA-seq data of the other three normal control kidney tissues were downloaded from the GEO database (GSE131685). R (version 4.1.2) was used for subsequent analysis. We applied the Merge function from Seurat R package (version 4.1.0) to merge the seven kidney datasets before integration and correction with the Harmony R package (version 0.1.0). Cells with gene expression < 200 or > 2500 or mitochondrial gene percentage > 30% were filtered according to the quality control standard. After that, 48573 high-quality kidney cells were kept for subsequent analysis. NormalizeData and ScaleData functions were applied to normalize and scale gene expression, after which top DEGs were selected using FindVariableFeatures for PCA analysis. The top 15 principal components and a resolution of 2 were applied for graph-based UMAP after clustering analysis. FindAllMarkers function was applied for DEGs calculation. GO and KEGG pathway enrichment analysis were implemented using clusterProfiler R package (version 4.2.2) for enriched signaling pathways. Monocle R package (version 2.22.0) was applied to perform pseudo time analysis. Cell-cell interaction analysis was performed by CellphoneDB.

## Data availability statement

The original contributions presented in the study are publicly available. This data can be found here: Gene Expression Omnibus, GSE241302.

## Ethics statement

The studies involving humans were approved by Ethics Institutional Review Board of Traditional Chinese Medicine Hospital of Kunshan. The studies were conducted in accordance with the local legislation and institutional requirements. The participants provided their written informed consent to participate in this study.

## Author contributions

The study was designed by MS, JL and YM. Patient screening and data investigation were performed by XC, CW, and YM. The data were analyzed by MS, YW, HZ and ZL. MS, YW, HZ, ZL, and JL drafted the paper. All authors contributed to the article and approved the submitted version.
