# Supplementary material for: Single-cell RNA sequencing shows the immune cell landscape in the kidneys of patients with idiopathic membranous nephropathy
Source: Front Immunol. 2023 Sep 4;14:1203062. doi: 10.3389/fimmu.2023.1203062 (PMC10507359; doi:10.3389/fimmu.2023.1203062)
Supplement: Supplementary file 1 [file DataSheet_1.docx]

**Supplementary files:**

**Table S1 |** Basic information of idiopathic membranous nephropathy patients and normal controls included in the study

| ﻿Donor (Study ID) | ﻿Group | ﻿Gender | ﻿Age (Years) | urine protein (-~4+) | microhematuria (/HP) | eGFR  (ml/min/1.73m2) | serum albumin (g/L) | Proteinuria (mg﻿/24h) | ﻿Pathological Diagnosis | ﻿PLA2R | ﻿Treatment | ﻿Remission |
| --- | --- | --- | --- | --- | --- | --- | --- | --- | --- | --- | --- | --- |
| mn5 | IMN | male | 36 | 4+ | 0 | 124.18 | 20.0 | 15.25 | IMN-II/III | + | steroid+ cyclosporine | CR |
| mn6 | IMN | male | 48 | 2+ | 2 | 107.79 | 28.8 | 4.35 | IMN-II | + | steroid+CTX | CR |
| mn7 | IMN | male | 37 | 4+ | 0 | 100.75 | 14.5 | 8.39 | IMN-II | + | steroid+CTX | CR |
| nc1 | normal control | male | 59 | - | 1 | NA | NA | NA | Renal clear cell carcinoma | NA | NA | NA |
| nc2 | normal control | male | 57 | NA | NA | NA | NA | NA | ﻿Papillary urothelial carcinoma of the renal pelvis | NA | NA | NA |
| nc3 | normal control | female | 59 | NA | NA | NA | NA | NA | Renal clear cell carcinoma | NA | NA | NA |
| nc4 | normal control | male | 65 | NA | NA | NA | NA | NA | Renal clear cell carcinoma | NA | NA | NA |

IMN: idiopathic membranous nephropathy; HP: high power field; eGFR, estimated glomerular filtration rate; PLA2R: phospholipase A2 receptor; CTX: cyclophosphamide; CR: complete remission; NA: not available.


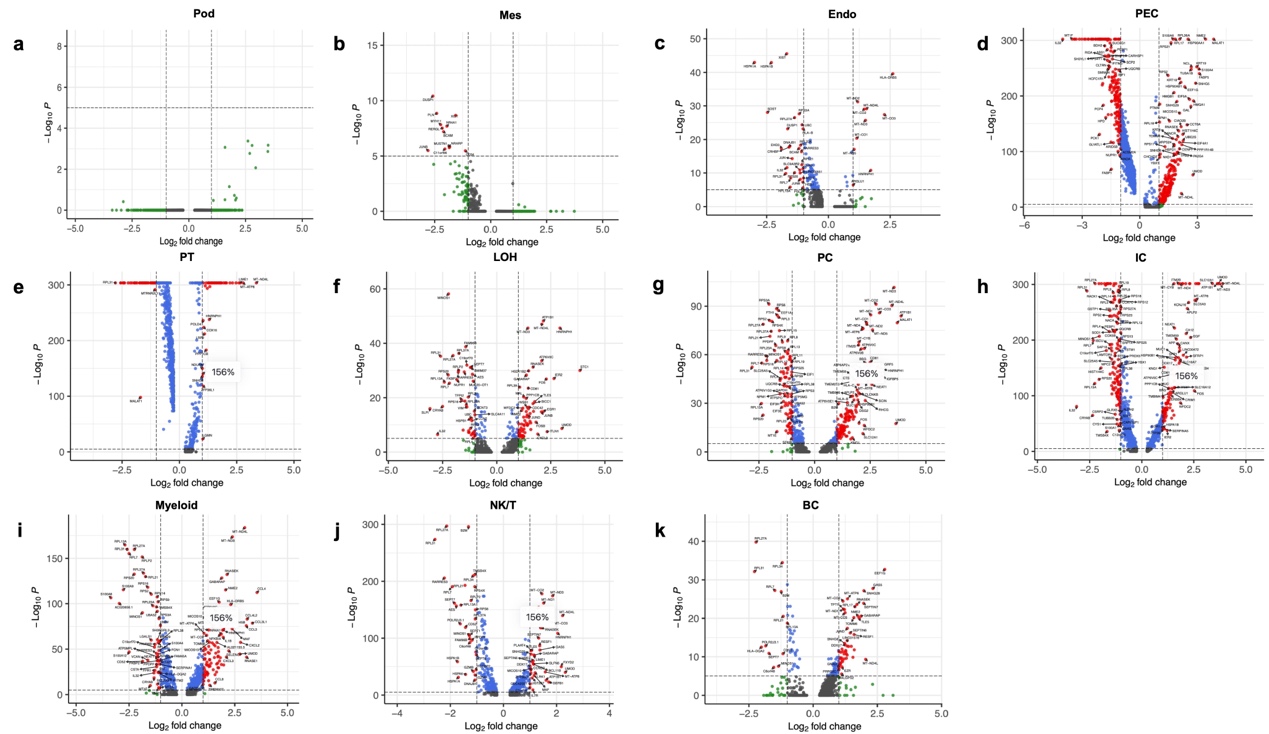


**Figure S1. Volcano plot showing the up- and down-regulated genes in (a) podocytes, (b) mesangial cells, (c) endothelial cells, (d) parietal epithelial cells, (e) proximal tubule cells, (f) loop of Henle, (g) principal cells, (h) intercalated cells, (i) myeloid cells, (j) NK/T cells, (k) B cells of IMN patients compared with control subjects.** Abbreviations: Pod, podocytes; Mes, mesangial cells; Endo, endothelial cells; PEC, parietal epithelial cells; PT, proximal tubule cells; LOH, loop of Henle cells; PC, principal cells; IC, intercalated cells; Myeloid, myeloid cells; NK/T, natural killer cells and T cells; BC, B cells.

**
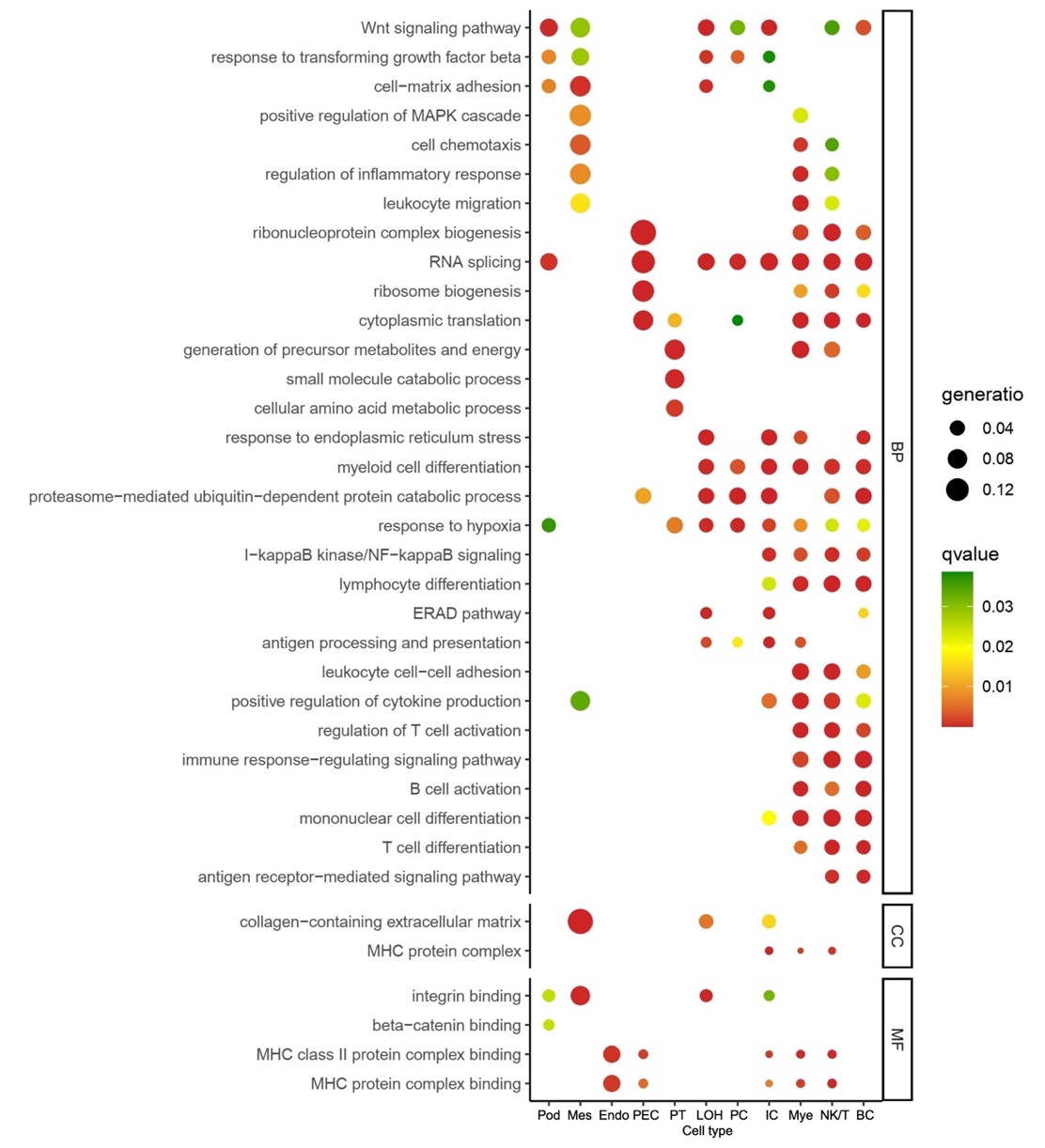
**

**Figure S2. Dot plot showing the GO enrichment analysis of different kidney cells.** Abbreviations: Pod, podocytes; Mes, mesangial cells; Endo, endothelial cells; PEC: parietal epithelial cells; PT, proximal tubule cells; LOH, loop of Henle; PC, principal cells; IC, intercalated cells; Myeloid, myeloid cells; NK/T, natural killer cells and T cells; BC, B cells.


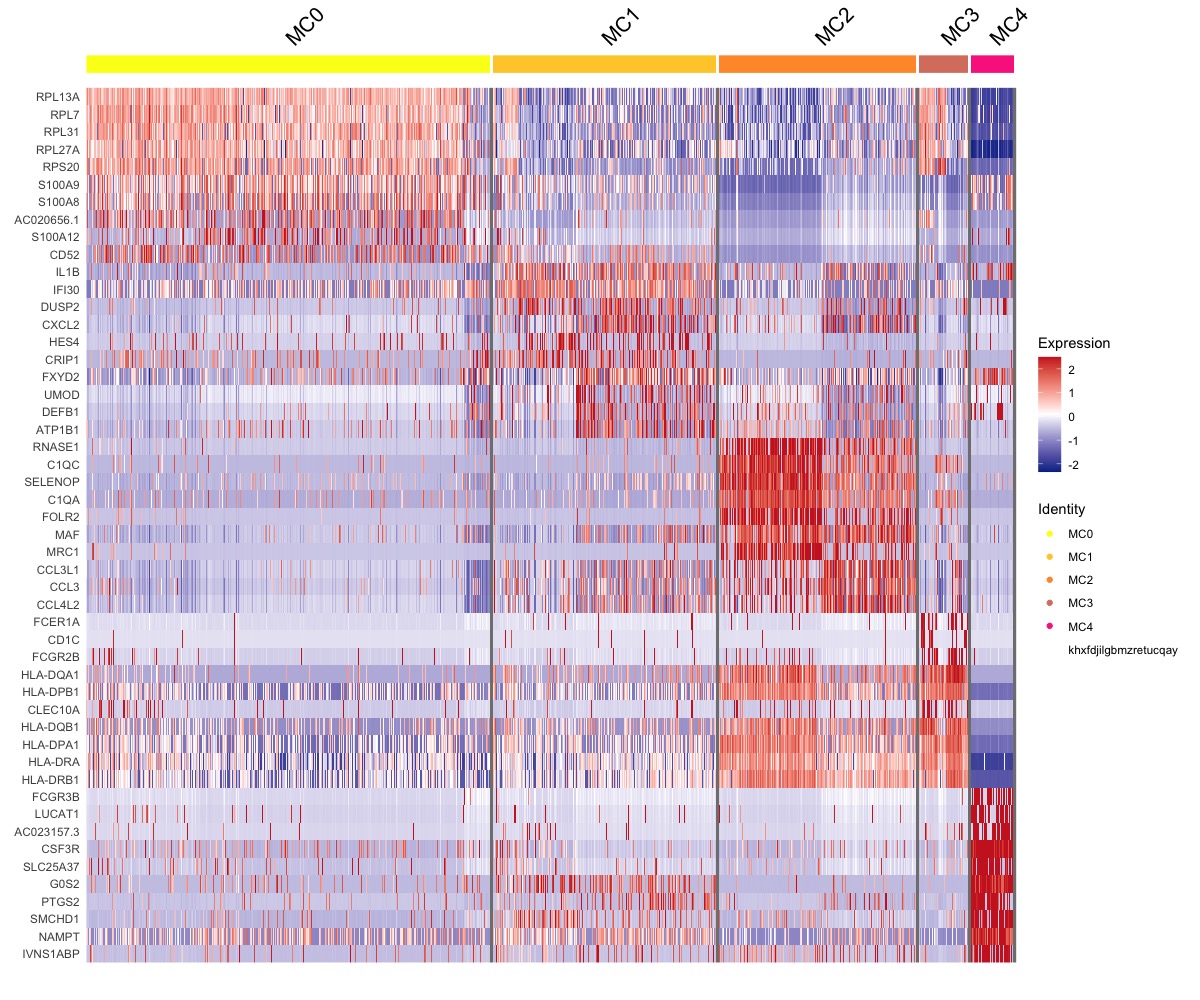


**Figure S3. Heatmap of kidney myeloid cells.** Five myeloid cell clusters were identified. The heatmap shows the expression of the top ten differentially up-regulated genes in each cluster.


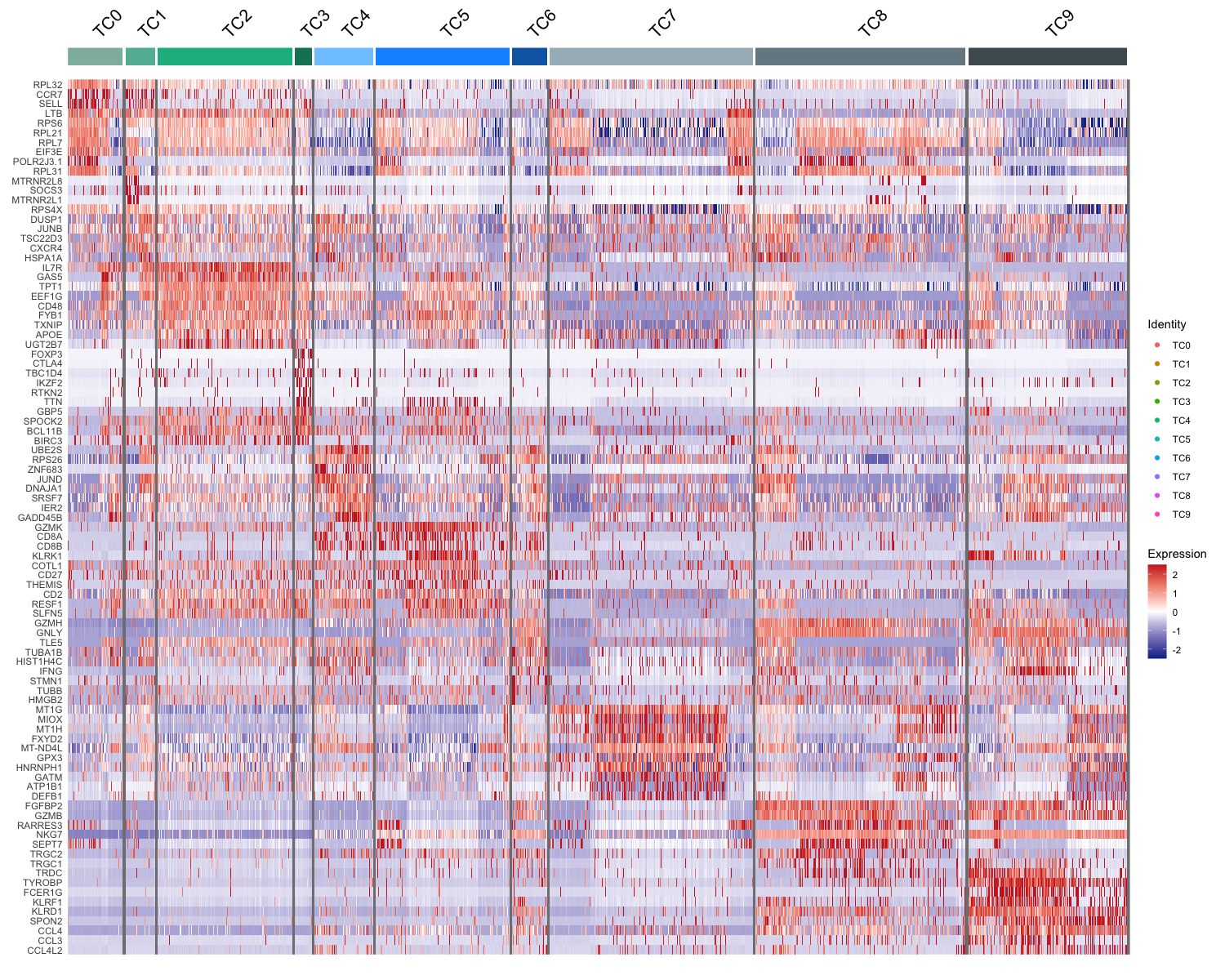


**Figure S4. Heatmap of kidney NK cells and T cells composed of ten subclusters.** The heatmap shows the expression of the top ten differentially up-regulated genes in each cluster.


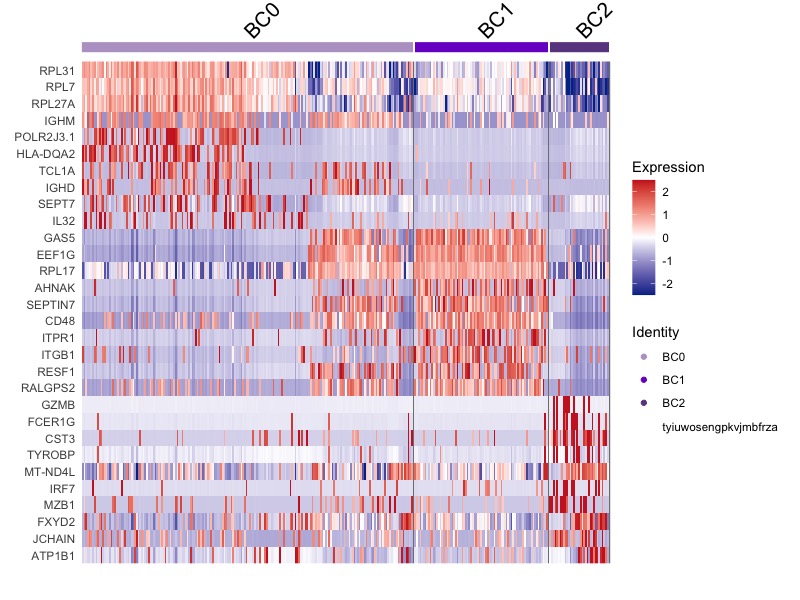


**Figure S5. Heatmap of kidney B cells composed of three subclusters.** The heatmap shows the expression of the top ten differentially up-regulated genes in each cluster.
